# Supplementary material for: Adaptation of Proteome and Metabolism in Different Haplotypes of Rhodosporidium toruloides during Cu(I) and Cu(II) Stress
Source: Microorganisms. 2023 Feb 22;11(3):553. doi: 10.3390/microorganisms11030553 (PMC10056549; doi:10.3390/microorganisms11030553)
Supplement: Supplementary file 1 [file microorganisms-11-00553-s001.zip › Supplementary_Materials.pdf]

Supplementary Materials: Adaption of Proteome and Metabolism in different haplotypes of *Rhodosporidium toruloides* during Cu(I) and Cu(II) stress

Table S3. OD_{600nm} of *R. toruloides* IFO0880 and IFO0559 grown in the presence of 0.5 mM Cu(I) and Cu(II) as well as controls (n=3).

IFO0880	Time [h]	0	6	24	30	48	53	71	76	96	102	119	125	144	148
0.03 % NH3	Mean	0.18	0.07	3.26	5.25	6.41	7.51	7.43	7.52	7.73		8.18	7.22	7.59	7.37
	SD	0.01	0.05	0.22	0.13	0.46	0.73	0.17	0.37	0.39		0.20	0.19	0.18	0.79
Cu(I)	Mean	0.20	0.10	0.14	0.24	0.22	0.21	0.56	1.23	3.50		5.80	5.60	6.18	5.81
	SD	0.01	0.02	0.06	0.07	0.07	0.13	0.57	1.59	1.68		1.22	0.88	0.39	0.63
0 mM	Mean	0.22	0.29	3.31	3.54	4.21	4.23	3.39	3.49	3.21	3.33	3.90	3.97	3.04	2.99
	SD	0.01	0.01	0.09	0.37	0.08	0.06	0.41	0.41	0.34	0.40	0.20	0.21	0.32	0.47
Cu(II)	Mean	0.24	0.23	2.79	3.94	5.27	5.37	5.97	5.97	5.90	5.90	5.89	5.57	5.79	5.49
	SD	0.01	0.02	0.24	0.23	0.25	0.21	0.41	0.41	0.47	0.66	0.73	0.55	0.95	0.93

IFO0559	Time [h]	0	6	24	30	48	53	71	76	96	102	119	125	144	148
0.03 % NH3	Mean	0.17	0.26	3.72	5.41	6.17	5.90	4.95	4.56	5.53		7.13	6.98	7.81	8.51
	SD	0.01	0.01	0.18	0.30	0.39	0.60	0.43	0.14	0.44		0.89	0.66	0.56	1.09
Cu(I)	Mean	0.19	0.15	0.24	0.55	1.13	1.68	3.27	4.06	5.23		5.13	5.07	5.55	4.37
	SD	0.01	0.01	0.06	0.10	0.20	0.57	0.42	0.20	0.19		0.11	0.09	0.06	0.32
0 mM	Mean	0.23	0.37	3.03	3.54	3.93	4.05	4.17	4.06	5.09	5.81	5.89	6.10	6.96	6.67
	SD	0.00	0.01	0.13	0.13	0.67	0.72	0.35	0.68	0.57	0.76	0.53	0.46	0.54	0.89
Cu(II)	Mean	0.23	0.25	2.34	3.83	5.76	5.93	6.89	7.15	7.61	8.15	7.93	7.87	8.83	8.75
	SD	0.01	0.02	0.54	0.32	0.66	0.64	0.90	0.69	1.00	0.85	0.86	0.91	0.97	0.86

Table S4. Dry cell weight (DCW) of of *R. toruloides* IFO0880 and IFO0559 grown in the presence of 0.5 mM Cu(I) and Cu(II) as well as controls (n=3) in g L^{-1}.

IFO0880

Mean	48 h	96 h	144 h
0.03% NH3	3.633	3.177	3.310
0.5 mM Cu(I)	0.911	2.163	3.053
0 mM	1.355	1.693	1.491
0.5 mM Cu(II)	1.998	2.657	2.329

SD	48 h	96 h	144 h
0.03% NH3	0.148	0.080	0.370
0.5 mM Cu(I)	0.067	0.418	0.117
0 mM	0.066	0.023	0.092
0.5 mM Cu(II)	0.350	0.287	0.452

IFO0559

Mean	48 h	96 h	144 h
0.03% NH3	3.130	2.980	4.060
0.5 mM Cu(I)	1.323	2.557	2.997
0 mM	1.962	3.013	3.069
0.5 mM Cu(II)	2.319	3.490	3.653

SD	48 h	96 h	144 h
0.03% NH3	0.114	0.369	0.236
0.5 mM Cu(I)	0.096	0.068	0.076
0 mM	0.199	0.176	0.069
0.5 mM Cu(II)	0.353	0.287	0.329

Table S5. Carotenoid accumulation of *R. toruloides* IFO0880 and IFO0559 grown in the presence of 0.5 mM Cu(I) and Cu(II) as well as controls (n=3) in Abs_{454nm} $mg^{-1}{}_{DCW}$.

IFO0880

Mean	48 h	96 h	144 h
0.03% NH3	0.037	0.052	0.078
0.5 mM Cu(I)	0.015	0.080	0.044
0 mM	0.043	0.045	0.056
0.5 mM Cu(II)	0.036	0.051	0.062

SD	48 h	96 h	144 h
0.03% NH3	0.005	0.005	0.012
0.5 mM Cu(I)	0.000	0.028	0.009
0 mM	0.011	0.013	0.005
0.5 mM Cu(II)	0.008	0.005	0.004

IFO0559

Mean	48 h	96 h	144 h
0.03% NH3	0.076	0.096	0.101
0.5 mM Cu(I)	0.086	0.064	0.120
0 mM	0.077	0.098	0.140
0.5 mM Cu(II)	0.081	0.116	0.120

SD	48 h	96 h	144 h
0.03% NH3	0.011	0.015	0.014
0.5 mM Cu(I)	0.015	0.011	0.015
0 mM	0.008	0.011	0.025
0.5 mM Cu(II)	0.012	0.007	0.006

Table S6. Lipid accumulation of *R. toruloides* IFO0880 and IFO0559 grown in the presence of 0.5 mM Cu(I) and Cu(II) as well as controls (n=3) in g $g^{-1}{}_{DCW}$.

IFO0880

Mean	48 h	96 h	144 h
0.03% NH3	0.162	0.253	0.193
0.5 mM Cu(I)		0.084	0.125
0 mM	0.057	0.046	0.052
0.5 mM Cu(II)	0.096	0.135	0.097

SD	48 h	96 h	144 h
0.03% NH3	0.064	0.022	0.048
0.5 mM Cu(I)		0.002	0.014
0 mM	0.022	0.022	0.016
0.5 mM Cu(II)	0.026	0.007	0.006

IFO0559

Mean	48 h	96 h	144 h
0.03% NH3	0.104	0.125	0.102
0.5 mM Cu(I)		0.138	0.164
0 mM	0.117	0.076	0.148
0.5 mM Cu(II)	0.122	0.156	0.170

SD	48 h	96 h	144 h
0.03% NH3	0.035	0.008	0.021
0.5 mM Cu(I)		0.012	0.038
0 mM	0.015	0.003	0.006
0.5 mM Cu(II)	0.012	0.005	0.020

Table S7. Fatty acid profile of of *R. toruloides* IFO0880 and IFO0559 grown in the presence of 0.5 mM Cu(I) and Cu(II) as well as controls (n=3) in mg g^{-1}_{DCW}.

IFO0880

	96 h	144 h	96 h	144 h	96 h	144 h	96 h	144 h
Mean	0.03% NH3		0.5 mM Cu(I)		0 mM		0.5 mM Cu(II)	
C13:0	0.000	0.000	0.000	0.000	0.077	0.041	0.008	0.016
C16:0	0.103	0.097	0.140	0.112	0.114	0.112	0.098	0.093
C17:00	0.052	0.059	0.024	0.046	0.002	0.004	0.056	0.048
C18:0	0.131	0.101	0.056	0.062	0.056	0.060	0.091	0.069
C18:1 (oleat)	0.456	0.455	0.434	0.501	0.471	0.476	0.433	0.445
C18:2	0.162	0.192	0.244	0.195	0.232	0.236	0.218	0.241
C18:3	0.022	0.023	0.064	0.024	0.040	0.039	0.031	0.033
C24:0	0.032	0.029	0.013	0.017	0.003	0.012	0.026	0.019
other	0.041	0.044	0.027	0.043	0.005	0.018	0.038	0.036

	96 h	144 h	96 h	144 h	96 h	144 h	96 h	144 h
SD	0.03% NH3		0.5 mM Cu(I)		0 mM		0.5 mM Cu(II)	
C13:0	0.000	0.000	0.000	0.000	0.053	0.021	0.001	0.002
C16:0	0.001	0.003	0.011	0.016	0.008	0.010	0.004	0.011
C17:00	0.002	0.001	0.013	0.029	0.003	0.004	0.004	0.008
C18:0	0.006	0.003	0.021	0.010	0.002	0.004	0.002	0.022
C18:1 (oleat)	0.009	0.010	0.081	0.033	0.032	0.007	0.015	0.044
C18:2	0.007	0.011	0.085	0.014	0.013	0.007	0.013	0.006
C18:3	0.001	0.001	0.039	0.002	0.002	0.003	0.001	0.002
C24:0	0.001	0.001	0.007	0.002	0.006	0.002	0.001	0.006
Rest	0.001	0.001	0.016	0.017	0.009	0.009	0.002	0.004

IFO0559

	96 h	144 h	96 h	144 h	96 h	144 h	96 h	144 h
Mean	0.03% NH3		0.5 mM Cu(I)		0 mM		0.5 mM Cu(II)	
C13:0	0.000	0.000	0.000	0.000	0.023	0.007	0.005	0.007
C16:0	0.080	0.119	0.136	0.145	0.118	0.130	0.139	0.148
C17:00	0.029	0.028	0.028	0.023	0.016	0.019	0.012	0.012
C18:0	0.037	0.090	0.105	0.086	0.091	0.100	0.103	0.102
C18:1 (oleat)	0.606	0.430	0.380	0.337	0.480	0.434	0.438	0.374
C18:2	0.162	0.231	0.213	0.260	0.200	0.225	0.212	0.249
C18:3	0.043	0.038	0.064	0.071	0.030	0.028	0.033	0.043
C24:0	0.010	0.030	0.036	0.038	0.022	0.027	0.029	0.033
other	0.034	0.035	0.038	0.039	0.021	0.032	0.030	0.032

	96 h	144 h	96 h	144 h	96 h	144 h	96 h	144 h

SD	0.03% NH3		0.5 mM Cu(I)		0 mM		0.5 mM Cu(II)	
C13:0	0.000	0.000	0.000	0.000	0.013	0.001	0.000	0.001
C16:0	0.002	0.010	0.001	0.004	0.003	0.002	0.002	0.003
C17:00	0.002	0.002	0.003	0.001	0.003	0.002	0.005	0.004
C18:0	0.004	0.010	0.005	0.006	0.004	0.003	0.009	0.009
C18:1 (oleat)	0.009	0.011	0.006	0.009	0.018	0.011	0.024	0.017
C18:2	0.004	0.006	0.005	0.011	0.012	0.010	0.008	0.003
C18:3	0.002	0.003	0.003	0.003	0.001	0.001	0.002	0.002
C24:0	0.002	0.001	0.002	0.002	0.003	0.000	0.001	0.002
Rest	0.001	0.003	0.001	0.002	0.009	0.002	0.001	0.001

Table S8. Differentially expressed proteins of zinc involved processes discussed here. For IFO0559 and IFO0880, fold change of samples grown under Cu(I) and Cu(II) stress was compared to respective control. Protein name and uniprot accession numbers are listed. Further fold change at 96 h and 144 h of samples are stated. Fractions below 1 depict downregulation. n.d. = not detected. up = upregulated below cut-off score. down = downregulated below cut-off score. Cut-off score: Significance ≤ 2 or fold change ≤ 2.

**IFO0559**						**IFO0880**					
		**Cu(I)**		**Cu(II)**				**Cu(I)**		**Cu(II)**	
	**Accession**	**96 h**	**144 h**	**96 h**	**144 h**		**Accession**	**96 h**	**144 h**	**96 h**	**144 h**
**zinc TFs und Enzymes**											
Zinc finger RING-type protein	M7Y0T6	n.d.	n.d.	7.14	n.d.	zinc finger C2H2-type domain containing protein	A0A0K3C855	up	n.d.	3.13	up
CCCH zinc finger DNA binding protein	M7XIP6	n.d.	n.d.	3.13	n.d.	zinc finger C2H2-type protein	A0A0K3CCX6	n.d.	down	2.78	n.d.
Zinc finger C2H2 LYAR-type protei	M7WU29	down	0.25	0.38	up	zinc finger PHD-type protein	A0A0K3CKU7	n.d.	n.d.	2.70	n.d.
Zinc_ribbon_15 domain-containing protein	M7X7S7	n.d.	n.d.	0.31	n.d.	Zn(2)-C6 fungal-type domain-containing protein	A0A2T0A8D7	n.d.	up	2.08	n.d.
Zinc finger C2H2-type Ino80 complex subunit Iec1	M7WMF2	n.d.	n.d.	2.22	2.69	C6 transcription factor	A0A0K3CFI9	n.d.	down	0.49	n.d.
Zinc-binding alcohol	M7X080	0.27	0.16	0.36	0.31	Zn(2)-C6 fungal-type domain-containing protein	A0A2S9ZZ37	down	n.d.	0.40	0.37
Zinc-type alcohol dehydrogenase	M7XS16	0.38	0.15	0.26	0.37	Zn(2)-C6 fungal-type domain-containing protein	A0A2T0AAD9	up	n.d.	0.33	16.67
C6 transcription factor	M7WWH8	n.d.	n.d.	0.46	n.d.	Zn(2)-C6 transcription factor	A0A0K3CBV3	n.d.	n.d.	0.25	0.43
Zn(2)-C6 transcription factor	M7WXN8	n.d.	n.d.	0.37	n.d.	Zn(2)-C6 fungal-type domain-containing protein	A0A2T0A4R7	down	down	0.14	n.d.
C6 transcription factor	M7WW97	n.d.	n.d.	0.31	3.49	Zn(2)-C6 fungal-type domain-containing protein	A0A2S9ZXU0	0.49	n.d.	0.06	0.33
C6 transcription factor	M7X4Q3	n.d.	n.d.	0.02	n.d.	zinc-binding oxidoreductase CipB	A0A0K3CAJ3	down	up	down	0.32
Zinc finger C2H2-type domain containing protei	M7XWE5	n.d.	n.d.	n.d.	0.08	Zn(2)-C6 fungal-type domain-containing protei	A0A2T0A0T5	up	up	up	2.08
Zinc-binding alcohol dehydrogenase	M7XH09	n.d.	n.d.	down	0.48	Zn(2)-C6 fungal-type transcription factor	A0A0K3C559	up	down	n.d.	0.49
Zinc finger CCCH-type protein	M7XWI2	n.d.	n.d.	n.d.	2.34	zinc finger RING-type domain containing protein	A0A0K3CHL8	2.54	up	down	down
Zinc finger PHD-type protei	M7WNZ6	n.d.	down	up	2.4	zinc finger RING-type protein	A0A0K3CNW0	3.53	up	up	n.d.

Zinc finger GATA-type protein transcription factor	M7WRP0	n.d.	up	n.d.	2.45
Zinc finger Mcm10/DnaG-type domain containing protein	M7X9P7	down	n.d.	n.d.	2.69
Zinc finger C2H2-type transcription factor	M7WJY8	n.d.	n.d.	n.d.	2.77
Zinc finger CCHC-type protei	M7XDT6	up	down	n.d.	3.75
Zinc finger FYVE-type protein	M7XRD7	0.23	0.46	n.d.	n.d.
Zinc-binding alcohol dehydrogenase	M7WHX0	2.82	3.09	down	n.d.
CCCH zinc finger and SMR domain containing protein	M7WTA3	0.32	n.d.	n.d.	down
Zinc finger RING-type protei	M7XDJ5	0.33	down	n.d.	n.d.
Zinc finger SWIM-type domain containing protein	M7WM85	0.38	n.d.	n.d.	n.d.
Zinc finger protein 622	M7XYS1	0.45	down	n.d.	n.d.
Zinc finger MYND-type domain containing protein	M7X5Z3	0.46	n.d.	n.d.	n.d.
Zinc finger C2HC5-type protein	M7WLL2	0.5	down	up	n.d.
Zinc metalloproteas	M7XLA9	2.03	up	down	n.d.
Zinc finger C2H2-type domain containing protein transcription factor (Fragment	M7WQB4	2.18	up	down	n.d.
Zinc-binding oxidoreductase CipB	M7XLX5	2.48	n.d.	n.d.	down
C6 transcription factor	M7X5A5	2.47	2.7	n.d.	n.d.
C6 transcription factor	M7X2I1	3.43	2.51	n.d.	n.d.
Zn(2)-C6 transcription factor	M7X050	0.3	down	down	n.d.
C6 transcription factor	M7WLE7	0.32	up	up	n.d.
Zn(2)-C6 fungal-type transcription factor	M7X108	0.41	n.d.	n.d.	n.d.
C6 transcription factor	M7WNV2	0.49	n.d.	n.d.	n.d.
Zn(2)-C6 transcription factor	M7X7T5	2.36	up	down	n.d.
Zn(2)-C6 fungal-type transcription factor	M7WTC8	2.41	up	down	down
Zn(2)-C6 fungal-type transcription factor	M7WUM2	2.62	up	down	down
C6 transcription factor	M7XMZ6	9.24	up	n.d.	n.d.
Zinc_ribbon_15 domain-containing protein	M7XCE4	n.d.	3.78	n.d.	up
AN1-type zinc finger protein	M7X6L0	n.d.	0.33	n.d.	down
Zinc finger Zim17-type	M7XHM6	down	0.18	down	down
Zinc finger protein nitric oxide synthase-interacting protein	M7X7K5	up	0.46	n.d.	up
Zinc finger HIT-type protein	M7XLY4	n.d.	2.47	n.d.	n.d.
Zinc finger RING-type domain containing protein	M7WXB4	n.d.	0.17	up	n.d.

zinc finger HIT-type protein	A0A0K3C6Z6	4.69	down	n.d.	n.d.
Zn(2)-C6 fungal-type domain-containing protein	A0A2T0AEP5	0.38	0.46	n.d.	n.d.
Zn(2)-C6 fungal-type domain-containing protei	A0A2T0AJ33	0.44	0.49	n.d.	up
Zn(2)-C6 fungal-type transcription factor	A0A0K3CBU4	4.61	3.33	up	up
Zn(2)-C6 fungal-type domain-containing protein	A0A2T0A2G9	0.39	n.d.	down	n.d.
Zn(2)-C6 fungal-type domain-containing protein	A0A2S9ZWV1	0.42	n.d.	down	n.d.
PHD-zinc-finger like domain-domain containing protein	A0A2T0AFB3	n.d.	2.77	n.d.	up
Zn(2)-C6 fungal-type domain-containing protein	A0A2S9ZZ34	n.d.	0.44	n.d.	n.d.
Zn(2)-C6 fungal-type transcription factor	A0A0K3C8D4	n.d.	2.01	n.d.	down
NADPH:quinone reductase Zn-dependent oxidoreductase	A0A0K3CDY3	n.d.	0.28	n.d.	n.d.

Zinc finger C2H2-type domain containing protein	M7XM09	n.d.	5.37	n.d.	n.d.
Zinc finger C2H2-type domain containing protein transcription factor	M7X1B9	n.d.	3.1	up	up
NADPH:quinone reductase Zn-dependent oxidoreductase	M7XTH0	8.18	4.48	n.d.	down

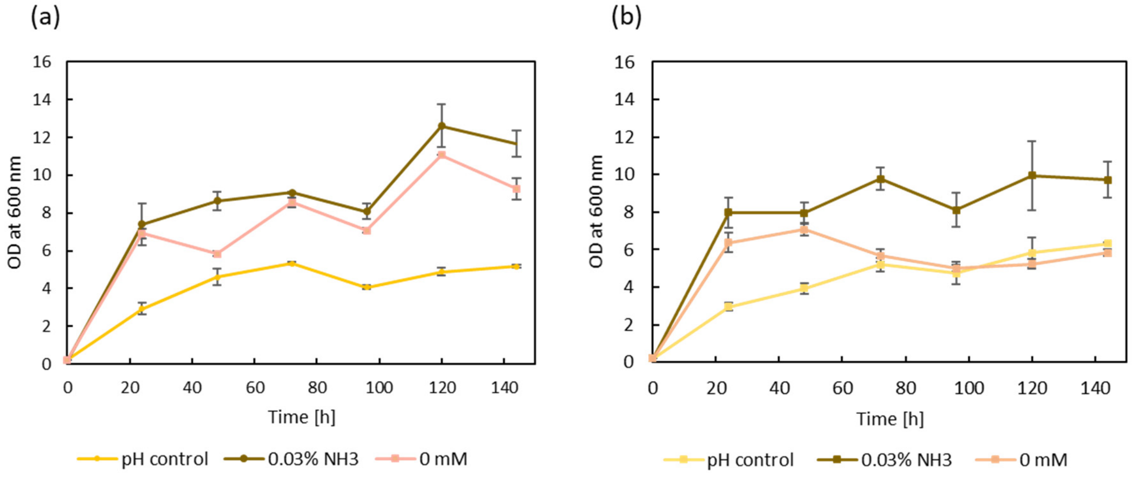

Figure S1. pH control in growth of haplotypes. Growth of *R. toruloides* in YNB (average pH 5.5), YNB supplemented with 0.03 % ammonia (average pH 7.2) and YNB adjusted to pH 7.2, for haplotypes IFO0559 (a) and IFO0880 (b).

IFO0559:

96 h: 0.03 % NH_3 vs 0.5 mM Cu(I)

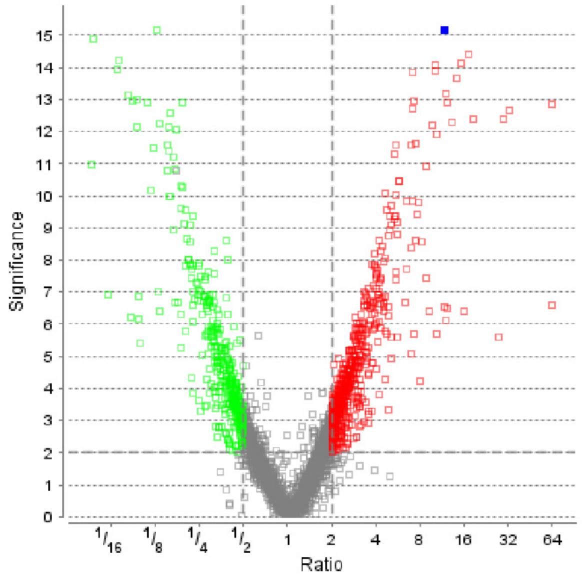

144 h: 0.03 % NH_3 vs 0.5 mM Cu(I)

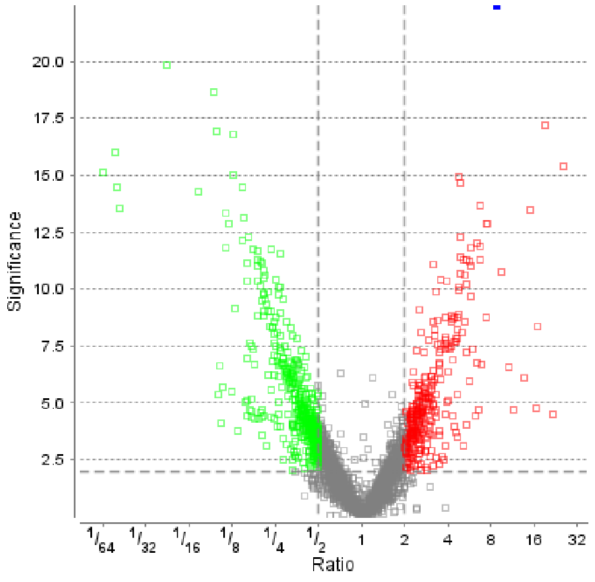

IFO0559:

96 h: 0.5 mM Cu(II) vs 0 mM

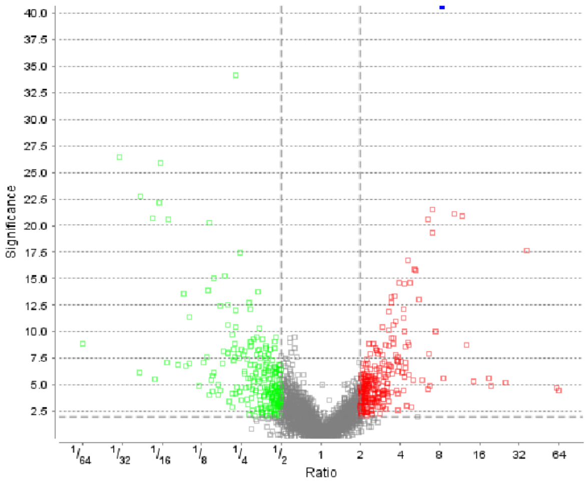

144 h: 0 mM vs 0.5 mM Cu(II)

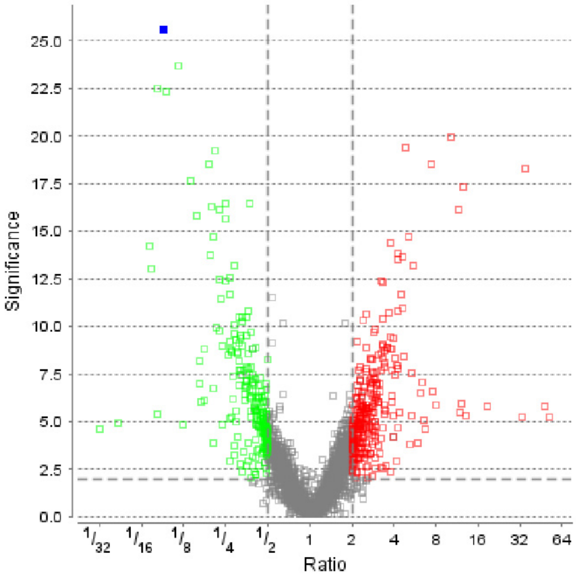

IFO0880:

96 h: 0.03 % NH_3 vs 0.5 mM Cu(I)

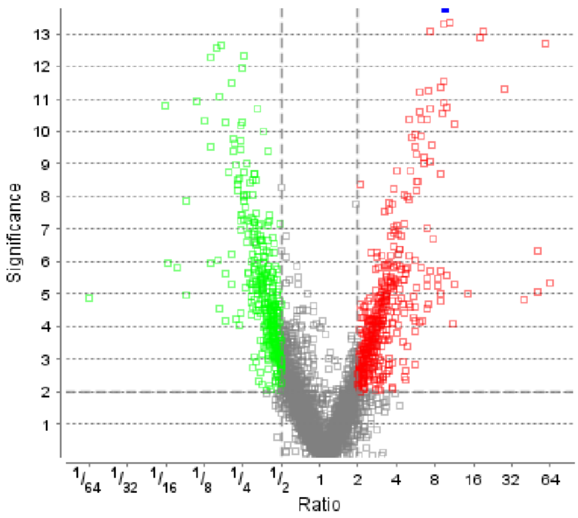

144 h: 0.03 % NH_3 vs 0.5 mM Cu(I)

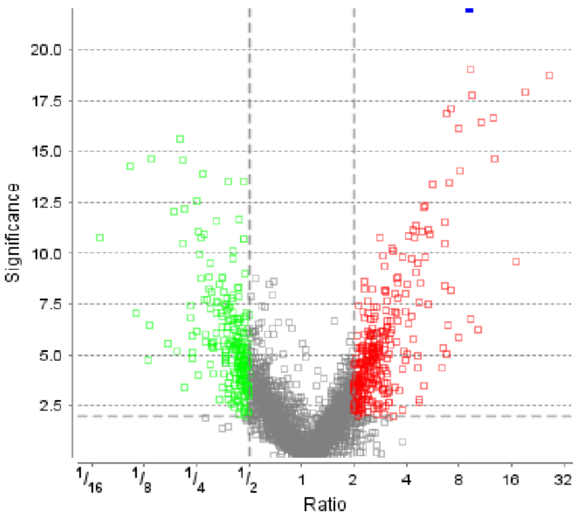

IFO0880:

96 h: 0.5 mM Cu(II) vs 0 mM

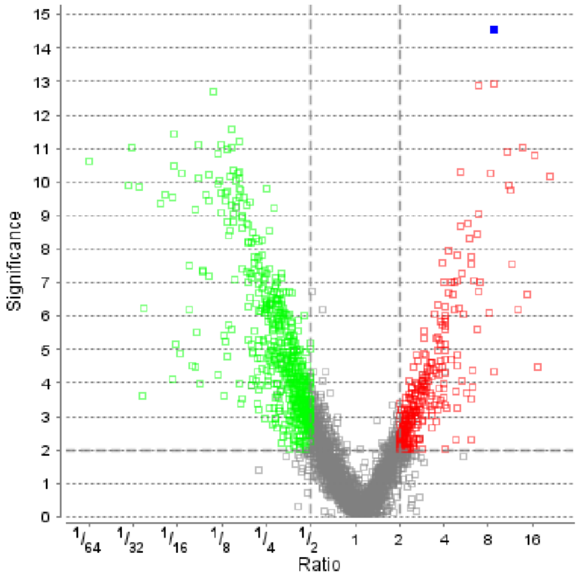

144 h: 0.5 mM Cu(II) vs 0 mM

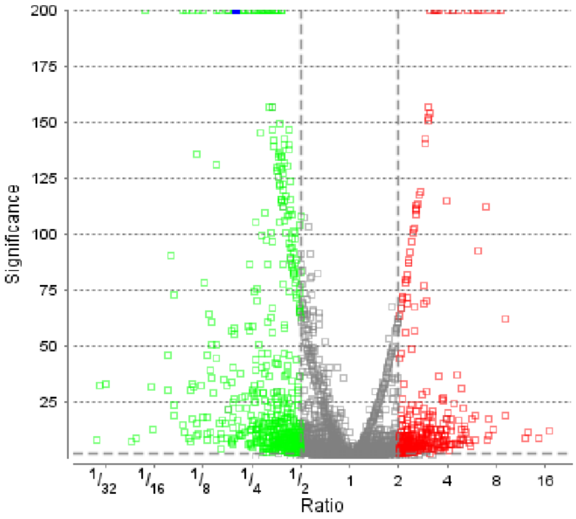

IFO0559: Ammonia supplementation

96 h: 0.03 % NH_3 vs 0 mM

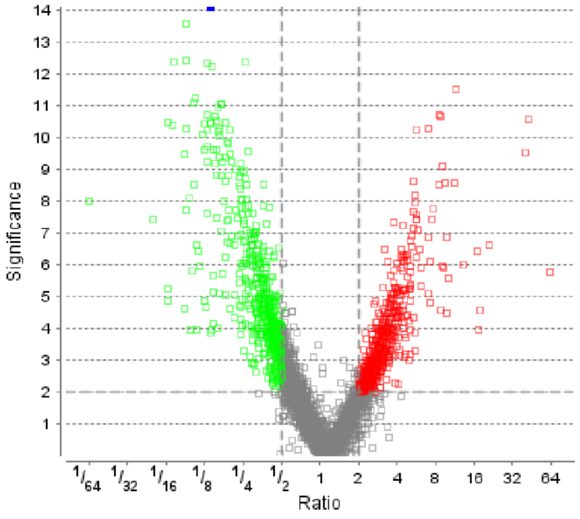

144 h: 0.03 % NH_3 vs 0 mM

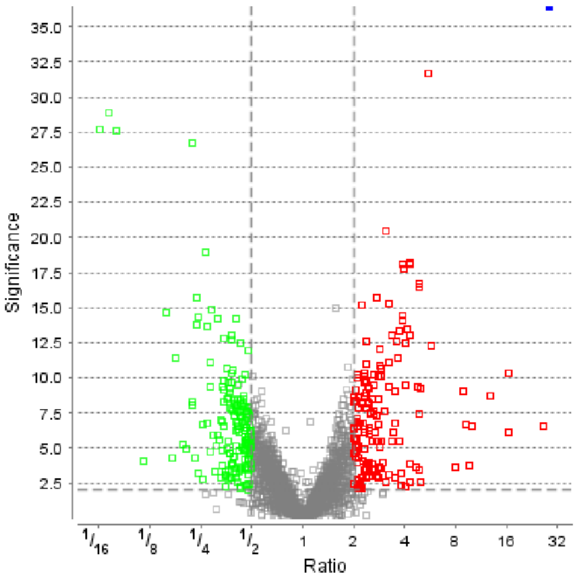

IFO0880: Ammonia supplementation

96 h: 0.03 % NH_3 vs 0 mM **144 h: 0.03 % NH_3 vs 0 mM**

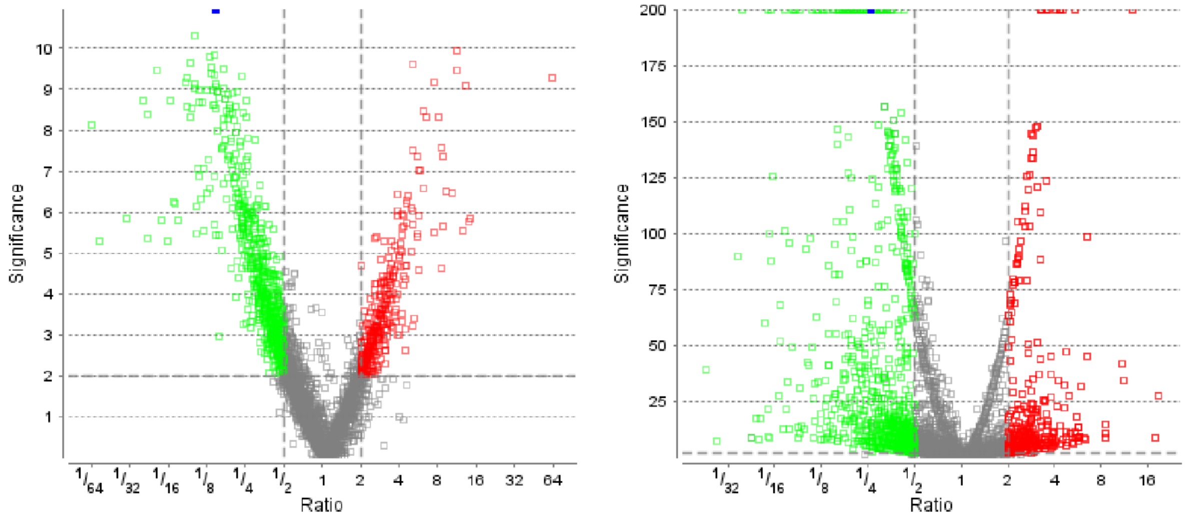

Figure S2. Volcano plots of the proteins quantified with significantly different abundance between samples grown with copper or ammonia supplementation compared to control. The significance is plotted against the Ratio. The non-axial vertical lines denote fold changes ≥ 2, while the non-axial horizontal lines denote a significance ≥ 2. All downregulated proteins are marked in green while upregulated proteins are marked in red. Figures compiled by PEAKS Studio Xpro.
